# A meta-analytic analysis of the acute effects of MDMA on empathy and emotion recognition in humans

**DOI:** 10.1038/s41598-025-25610-3

**Published:** 2025-11-29

**Authors:** Leehe Peled-Avron, Jacob S. Aday, Madeline M. Pantoni, Holly K. Hamilton, Joshua D. Woolley

**Affiliations:** 1 Department of Psychology, Bar-Ilan University, Ramat Gan, Israel; 2 Gonda Multidisciplinary Brain Research Center, Bar Ilan University, 5290002 Ramat Gan, Israel; 3 Department of Anesthesiology, University of Michigan, 500 S State St, Ann Arbor, MI USA; 4 University of California San Francisco, 505 Parnassus Ave, San Francisco, CA 94143 USA; 5 Department of Psychiatry and Behavioral Sciences, University of Minnesota School of Medicine, 420 Delaware St SE, Minneapolis, MN 55455 USA; 6 Minneapolis Veterans Affairs Health Care System, Minneapolis, MN USA; 7 Translational Psychedelic Research Program, Department of Psychiatry and Behavioral Sciences, Weill Institute for Neurosciences, University of California, San Francisco, CA USA; 8 San Francisco Veterans Affairs Medical Center, San Francisco, CA USA

**Keywords:** MDMA, Emotion, Empathy, Social cognition, Meta-analysis, Systematic review, Empathy, Human behaviour, Clinical pharmacology

## Abstract

3,4-methylenedioxymethamphetamine (MDMA) is an amphetamine derivative known as an “entactogen,” influencing emotional and social processing. Phase III clinical trials of MDMA-assisted psychotherapy for post-traumatic stress disorder have reported promising results, and MDMA-assisted therapy is currently under regulatory review. However, the precise mechanisms underlying positive treatment outcomes remain largely unknown. This meta-analysis aims to systematically synthesize existing data on the effects of MDMA on empathy and emotion recognition. Specifically, we focus on two established tasks, the Multifaceted Empathy Test (MET) and the Facial Emotion Recognition Task (FERT), to comprehensively evaluate MDMA’s role in social cognition. Separate meta-analyses for each emotion tested in the FERT will help discern potential variations in MDMA’s impact on specific emotional responses. Following PRISMA guidelines, we conducted the meta-analysis. The MET assesses cognitive and emotional empathy, while the FERT measures accuracy in identifying basic emotional expressions. Separate meta-analyses were performed for each emotion tested in the FERT. MDMA administration enhances emotional empathy but diminishes recognition accuracy of negative facial expressions (sadness, fear, anger). No significant effects on cognitive empathy or recognition of happy expressions were found. Understanding these nuanced effects may inform the optimization of therapeutic applications and considerations for safety in clinical settings. Further studies are warranted to elucidate the underlying psychological and neural mechanisms, emphasizing the importance of continued investigation into the multifaceted influence of MDMA on social cognition.

## Introduction

3,4-methylenedioxymethamphetamine (MDMA) is an amphetamine derivative and “entactogen”. Entactogens are a class of psychoactive drugs that can have profound effects on emotional and social processing by altering feelings of closeness, oneness, empathy, and emotion recognition^1^. In recent years, Phase III clinical trials of MDMA-assisted psychotherapy for post traumatic stress disorder (PTSD) have yielded large effect sizes and FDA approval of the treatment is anticipated as early as 2024 in the US^2^. Despite promising findings, the mechanisms that may underlie these treatment outcomes remain to be clarified. One hypothesis is that MDMA’s effects on emotional and social processing facilitate psychotherapy. That is, patients might be able to experience more openness and connection^3^, and as a result, might develop an improved therapeutic alliance with the therapist^4^. Moreover, MDMA’s effects relating to increased empathy, compassion, and decreased recognition of negative emotion may also be relevant mechanisms in treatment outcomes^5^. Yet, few studies to date have systematically analyzed the effects of MDMA on empathy and emotion recognition across studies. This meta-analysis addresses this important gap by synthesizing the currently available data on the effects of MDMA on empathy and emotion recognition.

Converging evidence from naturalistic reports, cross-sectional research, and clinical trials suggests that MDMA can affect empathy and emotion recognition^6^. First, a sample of polysubstance users characterized the distinctive effects of MDMA as being prosocial in nature, including increased empathy and gregariousness as well as lower interpersonal defenses, which could speculatively be related to decreased recognition of negative emotion^7^. Additionally, cross-sectional research suggests that during use, MDMA users exhibit differences in social cognition relative to non-users (e.g., increased cognitive empathy^8^, Practitioners have speculated that these social cognitive effects may have particular importance in a psychotherapeutic context^9^. For instance, it has been hypothesized that MDMA may particularly enhance the therapist‐patient emotional bond through social cognitive mechanisms; specifically, by reducing emotional responses to threat^10,11^, increasing disclosure in therapy, and enabling patients to receive both praise and criticism from others with more acceptance^12^. Collectively, these processes can improve the integrity of the therapeutic alliance, which is crucial to outcomes in all forms of psychotherapy^13^. More recently, MDMA clinical trials have begun measuring changes in social cognition through tasks designed to measure empathy and emotion recognition^3^, but this data has yet to be systematically aggregated. Nonetheless, the effects of MDMA on how one thinks about, and interacts with, others seem to be important to its clinical use.

In this meta-analysis, we focus on two tasks: the multifaceted empathy test (MET^14^; and the facial emotion recognition task (FERT^15^; These tasks garnered the highest number of studies, enabling a robust comparative analysis, furthermore, these tasks represent crucial functions in social cognition research. The MET is a commonly used test of empathy that uses photorealistic stimuli to enhance ecological validity compared to self-report questionnaires. It measures both cognitive and emotional empathy. Cognitive empathy is the ability to understand and perceive another person’s thoughts and feelings. It involves putting yourself in someone else’s shoes and seeing the world from their perspective. Cognitive empathy is a skill that can be learned and developed^16^. Emotional empathy is the ability to share and feel the emotions of another person. It is often described as “putting yourself in someone else’s shoes and feeling their pain.” Emotional empathy is a more innate ability, but it can also be strengthened over time^16^. Notably, the MET assesses emotional empathy through both explicit means (rating of empathic concern) and more implicit means (arousal ratings as a proxy for empathic concern) which we examine separately in this meta-analysis.

The FERT task measures the accuracy of identifying four facial expressions representing basic emotions (happiness, sadness, anger, and fear). The task uses pictures of faces that are morphed between 0% (neutral) and 100% of the emotion in 10% increments. Participants indicate the correct emotion displayed in each picture^17^. We performed a separate meta-analysis for each emotion for several reasons: First, it allows for a more nuanced understanding of how MDMA impacts specific emotional responses. MDMA may have differential effects on recognizing emotions like happiness, sadness, anger, and fear, and analyzing them separately helps to discern these distinctions. Second, studying emotions individually can reveal potential variations in the level of enhancement or impairment caused by MDMA for different emotions. For instance, MDMA might significantly improve recognition of happiness but have a minimal effect on recognizing fear—effects which might be lost when collapsing data across emotions.

Although the existing literature consistently indicates that MDMA can alter social cognition, the direction and magnitude of these effects have varied across studie, even within the same constructs. For example, several studies using the MET reported increased emotional empathy, particularly in explicit empathic concern^3,4^, whereas others found no significant effects on either explicit or implicit measures^18,19^. Findings on cognitive empathy have been even more inconsistent, with some studies showing small, non-significant increases^20,21^ and others showing no change at all. Similarly, results from studies employing the FERT have diverged: some report impaired recognition of negative facial expressions such as fear or anger^22,23^, whereas others observed no reliable change in recognition of sadness or happiness. These inconsistencies, evident even among studies with comparable designs, doses, and task parameters, underscore the need for a meta-analysis to determine the overall pattern and strength of MDMA’s effects on specific aspects of empathy and emotion recognition. The present study therefore systematically integrates results across these tasks and paradigms to clarify where the evidence converges or diverges.

This meta-analysis aims to address this gap in the literature by examining data from randomized controlled trials to determine the effects of MDMA on these social cognition functions.

## Methods

This study followed the PRISMA reporting guidelines for systematic reviews and meta-analysis^24^.

### Search strategy and inclusion criteria

From inception up to April 2025, a search for relevant articles was conducted in the PubMed (Medline), PsycINFO, and Scopus bibliographic databases using specific keywords: “mdma” OR “ecstasy” OR “3,4-methylenedioxymethamphetamine” AND “empathy” OR “emotion” OR “social cognition” OR “emotion recognition” OR “facial emotion recognition” OR “emotional empathy” OR “cognitive empathy” OR “social”. After a full text review, if a study was deemed eligible, its reference list was manually scrutinized for other relevant studies.

**Fig. 1:**
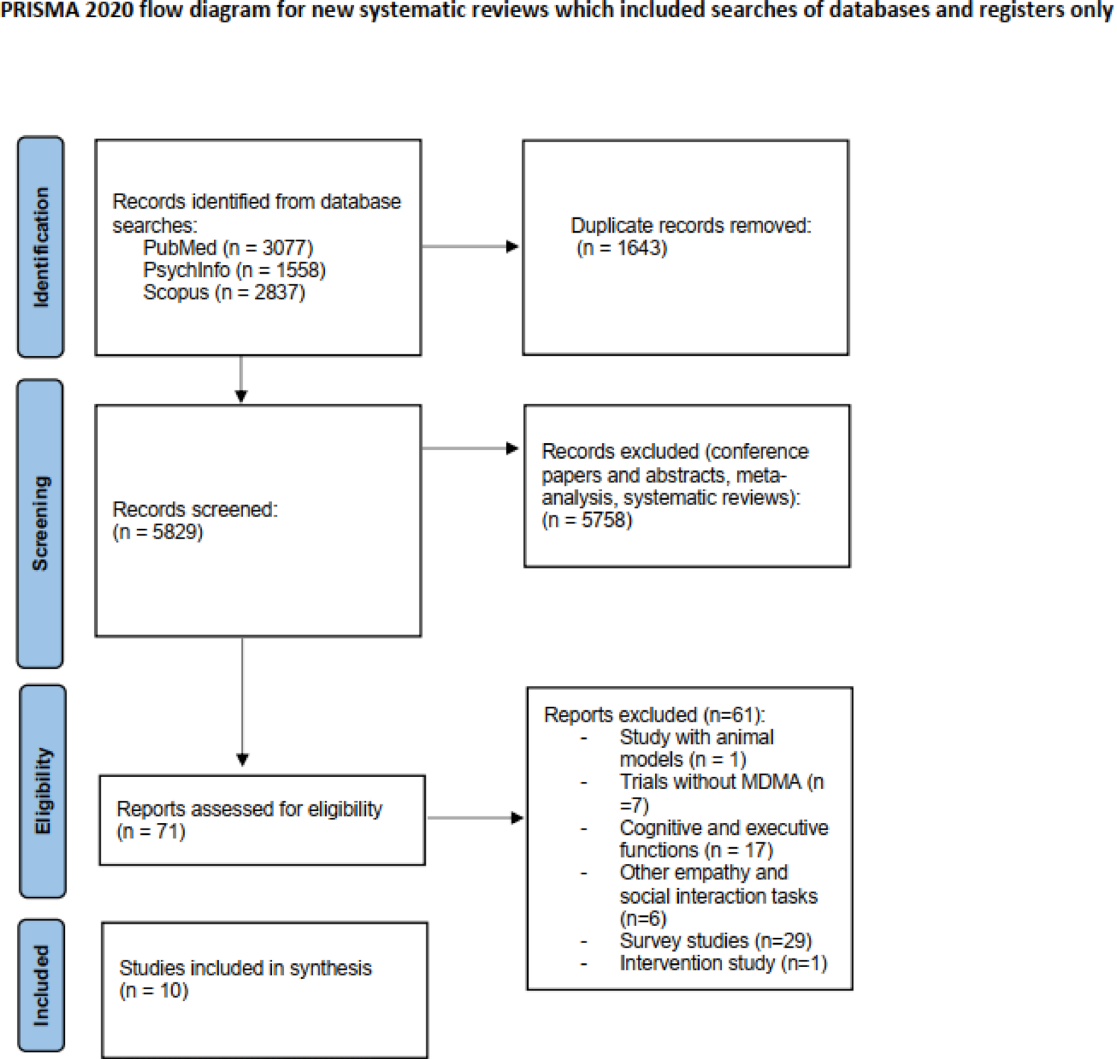
The search and selection procedure used to identify studies for inclusion in this meta-analysis.Template provided by PRISMA (www.prisma-statement.org). *Intervention studies include clinical populations which we did not examine here due to lack of data. We focused our meta-analysis on two specific tasks that received the highest number of studies. This decision was made after excluding cognitive tasks like memory or attention, as well as other social cognition tasks such as the ultimatum game, cyberball, and sexual behavior. This approach ensures a clear separation between non-social cognitive tasks and social cognition tasks that are outside the scope of our intended analysis.

Initially, duplicate records were eliminated, and subsequently, the titles and abstracts of the remaining publications were assessed. Any publications that clearly did not meet the inclusion criteria were excluded. The remaining publications were further evaluated by thoroughly reviewing the full text. The inclusion criteria were then applied again. To be considered for inclusion, studies had to meet the following eligibility criteria: (1) they had to be original, peer-reviewed, full-text articles written in English; (2) they had to involve healthy human participants; (3) they had to include a single-dose MDMA administration; (4) they had to include a placebo condition; (5) they had to be designed experimentally, with randomization, including randomized controlled trials (RCTs) in both crossover and parallel formats; and (6) they had to include an outcome(s) related to the FERT or MET tasks, provided that they met the eligibility criteria mentioned above. Studies were excluded if they were reviews and/or meta-analyses; included non-human subjects; assessed previous MDMA users retrospectively through the use of self-report questionnaires; or were letters, comments, abstracts, or conference papers. There were no exclusions based on the age or sex of the subjects, MDMA dose, administration routes, or study size. All included studies were randomized, double-blind, placebo-controlled experimental trials. We assessed methodological rigor, and all studies met basic criteria for low risk of bias with respect to randomization, blinding, and completeness of outcome reporting. Given the relatively small number of trials and their high methodological homogeneity, a formal risk-of-bias or GRADE assessment was not performed. No studies were excluded on the basis of methodological quality. A flowchart depicting the selection procedure is depicted in Fig. 1.

### Recorded measures and data extraction

All records retrieved through database searches were independently screened by two viewers (M.K and J.B) in accordance with PRISMA guidelines. Disagreements regarding study inclusion were discussed until consensus was reached. When necessary, a third viewer (L.A) was consulted to adjudicate unresolved cases. This multi-reviewer process was implemented to minimize selection bias and ensure reproducibility of the inclusion decisions. The recorded variables included general information, such as the study’s location, authors, year of publication, study design, tasks, number of subjects, and demographic variables such as age, gender, and MDMA dosage in milligrams (Table 1). For the calculation of effect sizes, means and standard deviations (SD) of the social cognition tasks were collected for both MDMA and control groups. If this information was not available in the publication or provided by the authors, effect sizes were estimated using one of the following methods: (1) by extracting mean and SD from published figures (using PlotDigitizer software, available at http://plotdigitizer.sourceforge.net), (2) based on F values, or (3) based on p values.

**Table 1:** demographic details on the included studies.

References	Sample size	m/f	Age—mean (s.d)	Age range	MDMA dosage
Bedi et al.^20^	21	12 M, 9F	24.4 (4.9)	18–38	0.75 and 1.5 mg/kg
Dolder^19^	24	12 M,12F	22.6 ± 3.0	19–29	125 mg
Gabay^21^	21	21 M	24.8 y, SD = 3.7	21–37	100 mg
Hysek^3^	32	16 M, 16F	25 3	20–31	125 mg
Hysek^3^	16	8 M, 8F	24.8 ± 2.6	–	125 mg
Kirkpatrick^23^	65	25F, 40 M	24.1 4.1	18 and 35	0.75 and 1.5 mg/kg
Kuypers^18^	20	12 M, 8F	21.60 (2.45)	18–26	75 mg
Schmid^5^	30	15 M, 15 W	24 ± 4.2	18–32	75 mg

### Statistical analyses

The meta-analyses were conducted using the ‘metafor’package^25^ in RStudio version 2022.07.2, and Comprehensive Meta-Analysis (Version 3.3.070). Hedges’g was utilized as the measure of effect size. Hedges’g corrects for sample size bias and is interpreted according to Cohen’s guidelines (0.2 for small effect, 0.5 for medium effect, and 0.8 for large effect). To evaluate heterogeneity across studies, Q statistics were used based on the random effects model, as significant heterogeneity is expected in meta-analyses of observational studies^26^. Q assesses the amount of observed dispersion, and a statistically significant Q suggests that the studies do not share a common true effect size. The I2 index was used to assess the proportion of total variability in effect size estimates, with I2 values of approximately 25%, 50%, and 75% indicating low, moderate, and high heterogeneity, respectively. Publication bias due to small study bias was quantitatively tested using Egger’s regression test^27^ for each outcome. An Egger’s test with a *P* value less than 0.05 was indicative of small study bias. Outlier and influence diagnostics were conducted to determine the impact of any outliers, and sensitivity re-analyses were performed without any identified outliers.

We gathered data for the MET task, which involved obtaining means and standard deviations for three variables: (1) cognitive empathy, which measured the accuracy in discerning emotions portrayed in the photos; (2) explicit emotional empathy, reflecting the participants’ratings indicating their level of empathy towards the person in the photo; and (3) implicit emotional empathy, indicating the arousal levels experienced while viewing the photos. As for the FERT task, we focused on accuracy rates in recognizing emotions such as happiness, sadness, fear, and anger. We conducted analyses to determine whether MDMA led to an increase or decrease in accuracy or ratings for each respective variable.

Several included studies used within-subject (crossover) designs, resulting in correlated observations between MDMA and placebo conditions. When available, within-subject statistics (means, standard deviations, and correlations) were used to compute standardized mean differences. When correlations were unreported, we used a conservative assumed correlation (r = 0.5) following recommendations for meta-analyses of psychopharmacological crossover studies^25,28^.

## Results

### MDMA’s effect on cognitive empathy

MDMA did not affect cognitive empathy compared to placebo (studies: n = 4, combined overall sample: *n* = 102, Hedges’g = 0.089, 95% CI from − 0.11 to 0.289, *p* = 0.381). Between studies heterogeneity was non-significant (Q = 0.473 [df = 3], I2 = 0, *p* = 0.924), thus meta-regressions were not performed.

**Fig. 2:**
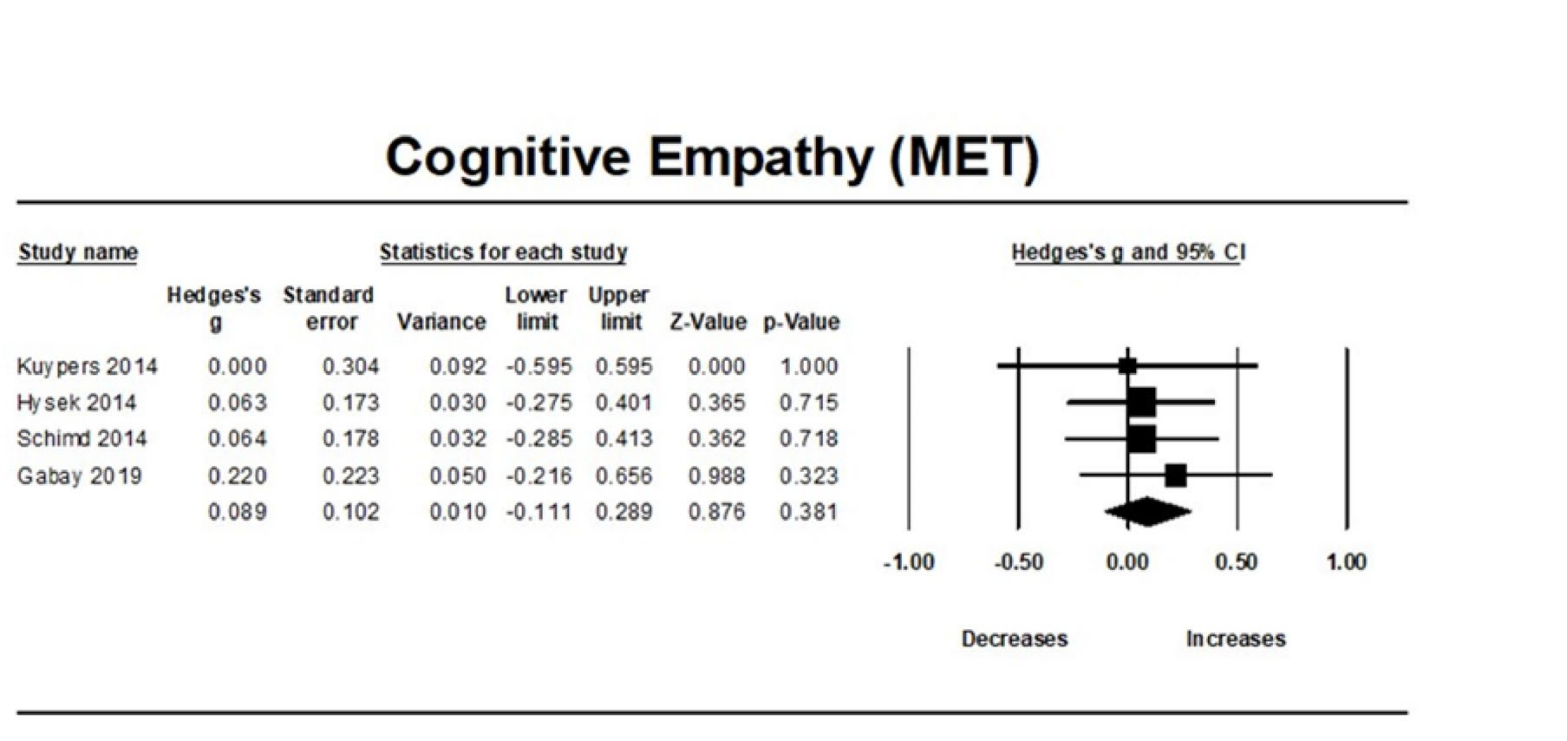
Forest Plot illustrating the influence of MDMA intervention on cognitive empathy scores measured by the MET task.

There was no significant publication bias due to small study effects (Egger’s regression test, *p* = 0.998). Moreover, the trim-and-fill analysis did not impute any potentially unpublished studies and the estimated effect size remained the same (observed effect size = adjusted effect size = 0.089). Influence diagnostics did not identify any outliers (Fig. 2).

### MDMA’s effect on explicit emotional empathy

MDMA was found to improve explicit emotional empathy compared to placebo (N = 4, overall sample = 102, Hedge’s g = 0.288, 95% CI from 0.094 to 0.481, *p* = 0.003). Between studies heterogeneity was non-significant (Q = 1.599 [df = 3], I2 = 0, *p* = 0.6595), thus meta-regressions were not performed.

Egger’s regression test (*P* = 0.438) found no significant publication bias due to small study effects.

**Fig. 3:**
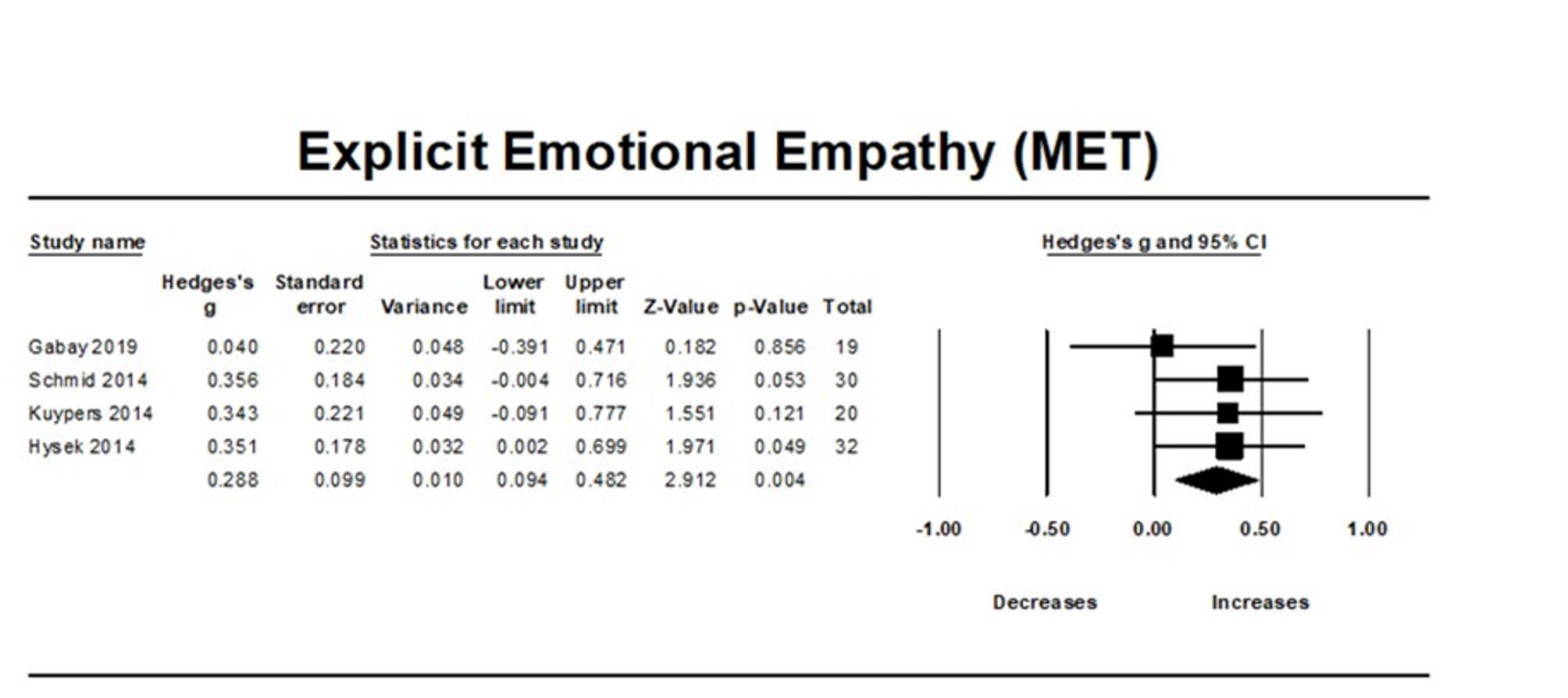
Forest Plot illustrating the influence of MDMA intervention on explicit emotional empathy scores measured by the MET task.

Moreover, the trim-and-fill analysis did not impute any potentially unpublished studies and the estimated effect size remained the same (observed effect size = adjusted effect size = 0.288). Influence diagnostics did not identify any outliers (Fig. 3).

### MDMA’s effect on implicit emotional empathy

MDMA was found to improve implicit emotional empathy compared to placebo (N = 3, overall sample = 82, Hedge’s g = 0.228, 95% CI from 0.015 to 0.442, *p* = 0.035). Between studies heterogeneity was non-significant (Q = 0.599 [df = 2], I2 = 0, *p* = 0.741), thus meta-regressions were not performed.

Egger’s regression test (*P* = 0.759) was conducted, and it found no significant publication bias due to small study effects.

**Fig. 4:**
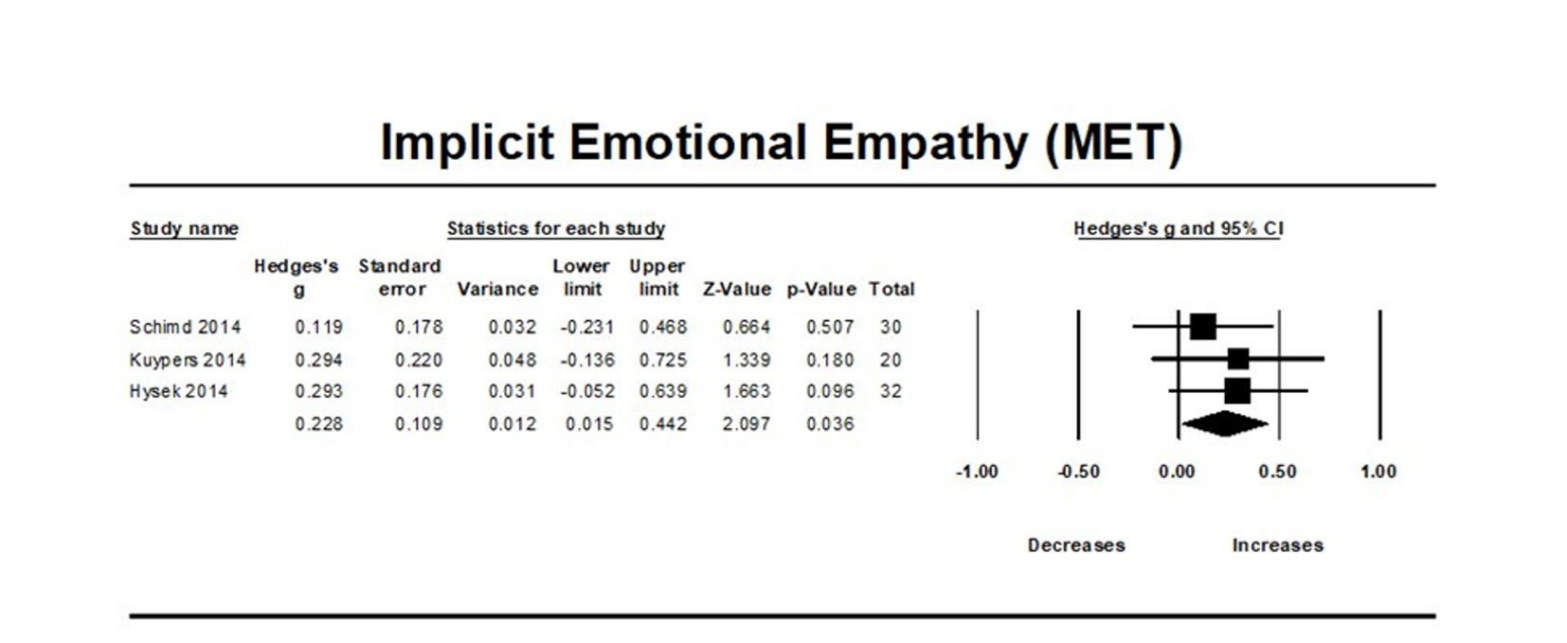
Forest Plot illustrating the influence of MDMA intervention on implicit emotional empathy scores measured by the MET task.

Moreover, the trim-and-fill analysis did not impute any potentially unpublished studies and the estimated effect size remained the same (observed effect size = adjusted effect size = 0.288). Influence diagnostics did not identify any outliers (Fig. 4).

### MDMA’s effect on facial emotion recognition—happy

MDMA did not affect recognition of happy facial expressions compared to placebo (n = 9, overall sample = 294, Hedges’g = -0.035, 95% CI from − 0.614 to 0.076, *p* = 0.536). Between studies heterogeneity was non-significant (Q = 2.872 [df = 8], I2 = 0, *p* = 0.9421), thus meta-regressions were not performed.

**Fig. 5:**
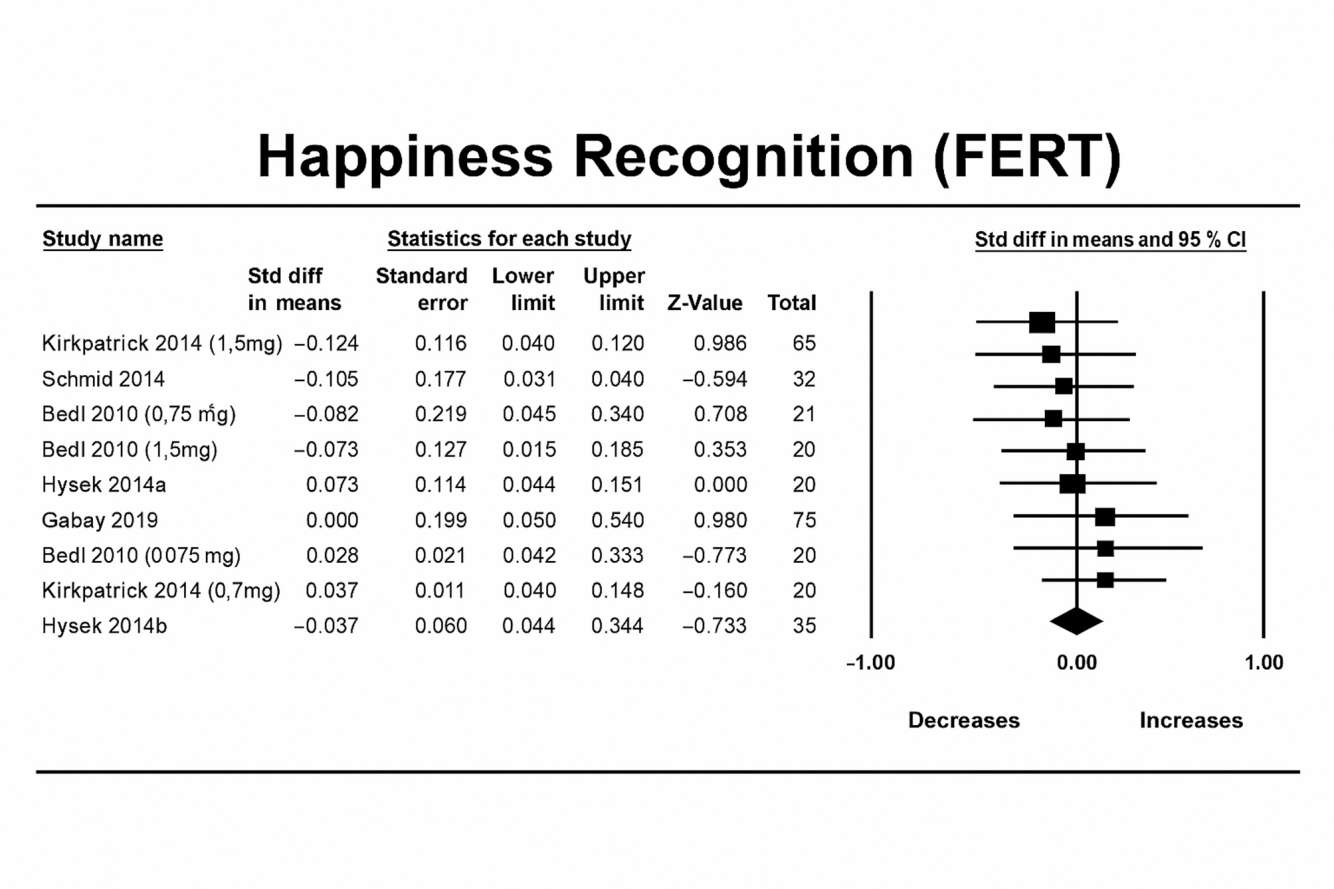
Forest Plot illustrating the influence of MDMA intervention on facial emotion recognition of happiness scores measured by the FERT task.

Egger’s regression test was conducted (*p* = 0.4016) and demonstrated no significant publication bias due to small study effects. Moreover, the trim-and-fill analysis did not impute any potentially unpublished studies and the estimated effect size remained the same (observed effect size = adjusted effect size = − 0.035). Influence diagnostics did not identify any outliers (Fig. 5).

### MDMA’s effect on facial emotion recognition—sad

Initially, MDMA was not found to affect recognition of sad facial expressions compared to placebo (n = 9, overall sample = 294, Hedges’g = − 0.104, 95% CI from − 0.228 to 0.0192, *p* = 0.097). Between studies heterogeneity was non-significant (Q = 8.005 [df = 8], I2 = 14.12%, *p* = 0.432), thus meta-regressions were not performed.

Egger’s regression test was conducted (*P* = 0.231) and demonstrated no significant publication bias due to small study effects. A trim-and-fill analysis identified two studies as potentially affecting the findings and imputation of these studies resulted in an adjusted effect size of − 0.058, *p* = 0.338 (observed effect size = − 0.104, *p* = 0.09). Influence diagnostics identified one potential outlier.

**Fig. 6:**
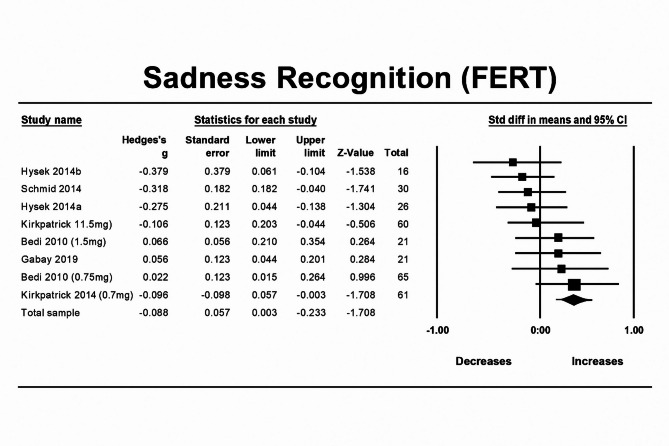
Forest Plot illustrating the influence of MDMA intervention on facial emotion recognition of sadness scores measured by the FERT task.

A sensitivity analysis, which involved re-analysis without the identified outlier, revealed a significant effect of MDMA on recognition of sad facial expressions, meaning that MDMA decreased sadness recognition in facial expressions (n = 8, overall sample = 229, Hedges’g = − 0.159, 95% CI from − 0.286 to − 0.032, *p* = 0.0139) (Fig. 6).

### MDMA’s effect on facial emotion recognition—fear

MDMA decreased recognition of fearful facial expressions compared to placebo (N = 9, overall sample = 294, Hedge’s g = − 0.239, 95% CI from − 0.402 to − 0.077, *p* = 0.0038). Between studies heterogeneity was non-significant (Q = 14.817 [df = 8], I2 = 0, *p* = 0.0628), thus meta-regressions were not performed.

Egger’s regression test was conducted and resulted in a *P* value of 0.189 demonstrating no significant publication bias due to small study effects.

**Fig. 7:**
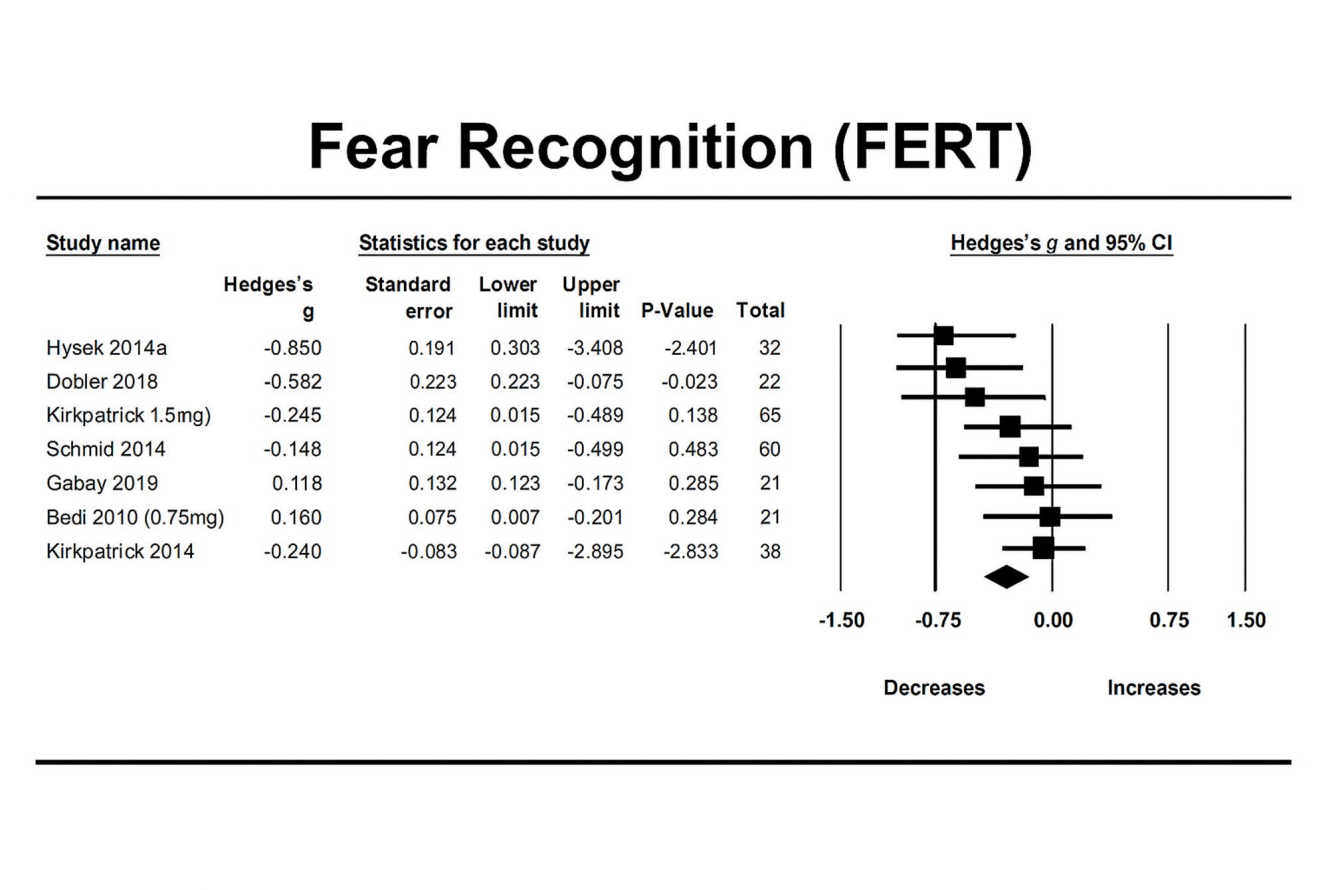
Forest Plot illustrating the influence of MDMA intervention on facial emotion recognition of fear scores measured by the FERT task.

A trim-and-fill analysis identified one study as potentially affecting the findings and imputation of this study resulted in an adjusted effect size of − 0.192, *p* = 0.034 (observed effect size = − 0.239, *p* = 0.0038). Influence diagnostics identified one potential outlier. A sensitivity analysis, which involved re-analysis without the identified outlier, revealed a similar significant effect of MDMA on recognition of fearful facial expressions (n = 8, overall sample = 278, Hedges’g = − 0.177, 95% CI from − 0.314 to − 0.0404, *p* = 0.0111) (Fig. 7).

### MDMA’s effect on facial emotion recognition—anger

MDMA decreased recognition of angry facial expressions compared to placebo (n = 9, overall sample = 294, Hedges’g = − 0.227, 95% CI from − 1.189 to 0.141, *p* < 0.001). Between studies heterogeneity was significant and high (Q = 45.439 [df = 8], I2 = 96.59%, *p* < 0.0001).

Egger’s regression test was found to be significant (*p* < 0.0001) demonstrating significant publication bias due to small study effects.

**Fig. 8:**
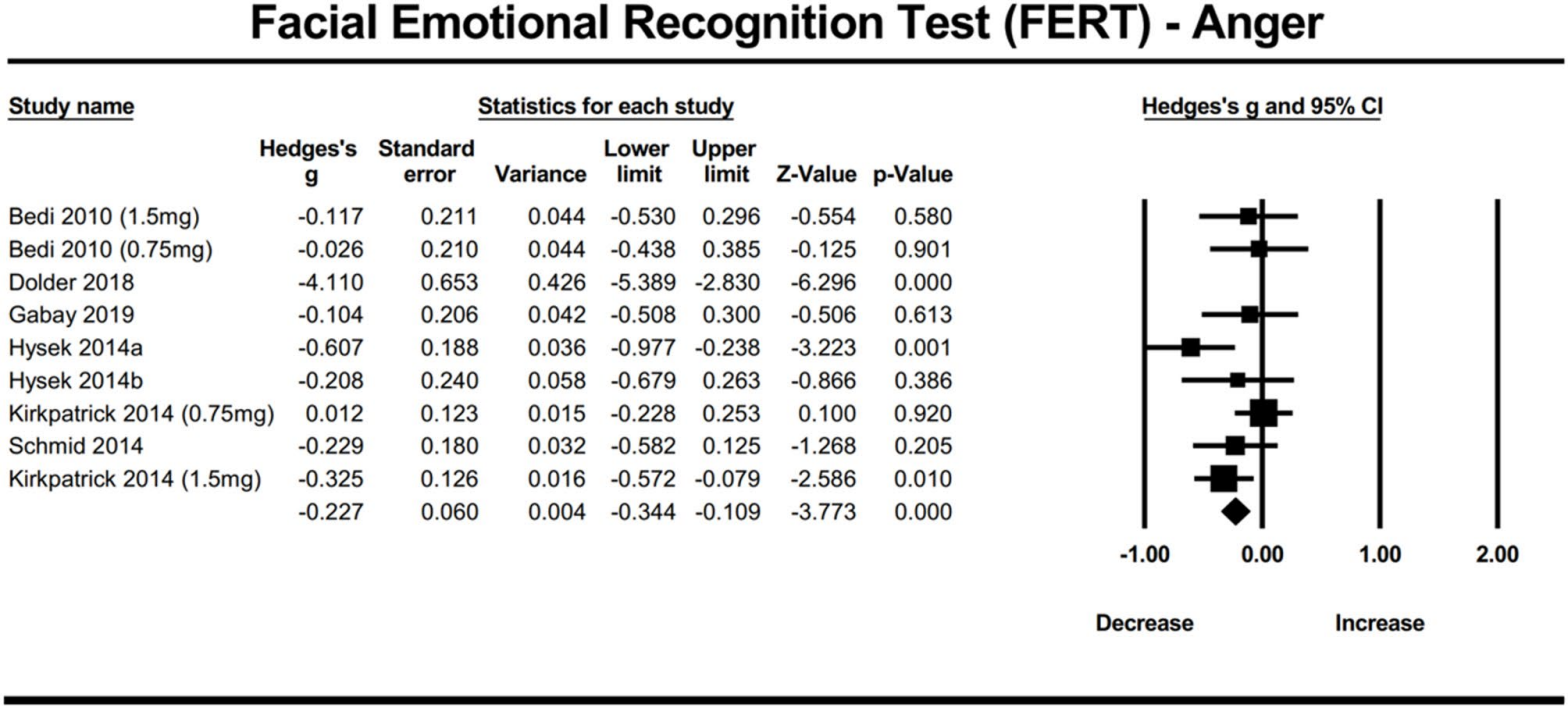
Forest Plot illustrating the influence of MDMA intervention on facial emotion recognition of anger scores measured by the FERT task.

Moreover, the trim-and-fill analysis did not impute any potentially unpublished studies and the estimated effect size remained the same (observed effect size = adjusted effect size = 0.523). However, influence diagnostics identified one potential outlier. A sensitivity analysis, which involved re-analysis without the identified outlier, revealed that MDMA continued to significantly reduce the recognition of angry facial expressions (n = 8, overall sample = 270, Hedges’g = − 0.198, 95% CI from − 0.347 to − 0.05, *p* = 0.0088). Between studies heterogeneity was reduced and was non-significant (Q = 9.738 [df = 7], I2 = 32.20%, *p* = 0.203). Egger’s regression test resulted in a non-significant *P* value of (*p* = 0.902) demonstrating that without the one study, there is no significant publication bias due to small study effects (Fig. 8).

## Discussion

This study presents a meta-analysis of the currently available data on the acute effects of MDMA on social cognition functions, specifically cognitive and emotional empathy and emotion recognition from facial expressions. The findings may indicate that, when compared to placebo, MDMA increases both explicit and implicit emotional empathy, while reducing the recognition accuracy of negative facial expressions including sadness, fear, and anger. In contrast, MDMA was not found to affect cognitive empathy or the recognition of happy facial expressions.

To the best of our knowledge, this is the first meta-analysis on the impact of MDMA on emotion recognition using the MET and the first using the FERT. We chose to focus on just one task for emotion recognition rather than including studies which used other tasks such as the “Reading the mind in the eyes task” (RMET)^29^ for emotion recognition. Moreover, we could only find one study that used the RMET task^30^ and therefore could not perform a meta-analysis on the effects of MDMA on emotion recognition using the RMET task. Focusing on results from just one task across different studies in the meta-analysis is advantageous for several reasons: (1) the analysis yields a higher level of methodological consistency across studies and this consistency minimizes the potential for confounding variables or differences in task designs (for example recognizing emotions from a whole facial expression as opposed to the eye region), leading to a more precise and reliable estimation of the effect size; (2) a meta-analysis on a single task allows for a more direct comparison of results from various studies, as they all share a common measurement approach; (3) a single-task meta-analysis allows researchers to gain deeper insights into the specific factors or variables related to that particular task (for example the four different emotions that were examined in the FERT) and its relationship to the outcome of interest—the effect of MDMA on emotion recognition, facilitating a more nuanced understanding of the phenomenon under investigation. Thus, examining the effect of MDMA on emotion recognition using the FERT allows us to discern whether MDMA increases or decreases specific emotions, leading to a more nuanced exploration of this phenomenon. As human emotions are multifaceted, a more complex approach is needed to explore this topic.

### MDMA’s effects on empathy

MDMA was found to increase emotional empathy but not cognitive empathy. Our findings are consistent with and extend prior work by Kuypers et al.^31^, who conducted a pooled subjects study to examine the effect of MDMA on empathy in this group. Our meta-analysis extends upon their earlier work by incorporating a new study published in 2019^32^. Moreover, our meta analysis is different from a pooled subjects study since in a pooled study, researchers combine individual participant data from multiple studies into a single dataset, allowing for the analysis of larger sample sizes and enhancing statistical power. On the other hand, a meta-analysis such as we did involves the systematic review and statistical synthesis of results from multiple independent studies. This enabled us to draw more generalized conclusions, increasing the robustness of the findings and identifying patterns across different studies. The effect of MDMA on emotional empathy is believed to be primarily attributed to the substance’s ability to augment the release of oxytocin, yet this conception is complex.

Oxytocin is a hormone that acts as a neuromodulator and plays a role in social behaviors in both animals and humans^33^. Some studies indicate that oxytocin can promote affiliative behaviors such as generosity, empathy, and altruism^34–36^. It has been observed that MDMA increases oxytocin levels^37^. As a result, the elevation of emotional empathy associated with MDMA use might be attributed to the rise in oxytocin levels, which in turn facilitates prosocial behaviors. However^18^, did not find significant increases in peripheral oxytocin levels following MDMA administration. Furthermore, their study indicated that increased peripheral oxytocin levels did not enhance emotional empathy, nor did it correlate with the increase in emotional empathy following MDMA use. Additionally, contrary to the belief that oxytocin exclusively promotes positive social emotions, evidence suggests that oxytocin can also enhance negative social emotions, such as aggression towards rival out-group members^38^ and envy^39^. Consequently, an alternative hypothesis has been proposed, suggesting that oxytocin amplifies the salience of social cues, and its effects are contingent upon individual characteristics and the specific situation in which it is administered^40,41^. This perspective shares similarities with the set and setting hypothesis^42^ of psychedelic therapies, which posits that the effects of psychedelic substances are heavily influenced by the mindset of the individual and the contextual setting in which they are taken^43,44^). Future research should elucidate the right set and setting for the safe use of MDMA.

### MDMA’s effects on emotion recognition

This is the first meta analysis to explore the effect of MDMA on facial emotion recognition. Overall, our results suggest that MDMA acutely reduces the ability to identify sad, fearful and angry facial expressions. These results could be explained by the effects of MDMA on amygdala activity. In an fMRI study by^22^, acute MDMA administration led to a reduction in amygdala reactivity specifically towards angry facial expressions relative to neutral faces. Additionally, a meta-analysis investigating long-term MDMA users revealed significant reductions in serotonin transporter density in the amygdala^45^. Another study utilizing 18FFlourodeoxyglucose PET to examine resting brain metabolism found pronounced reductions in left amygdala as well as the caudate and putamen in MDMA users compared to controls^46^. Collectively, these findings suggest that MDMA administration is associated with diminished amygdala activity, potentially contributing to the altered emotional processing and recognition of negative facial expressions seen in the literature.

It has been suggested that MDMA-induced-decreases in recognizing negative emotions may at least partly explain the enhanced inclination for social interaction typically seen after ingestion of MDMA^47^. People in the user’s surroundings become less scary or even just less negative, therefore this can facilitate approach behaviors. This effect could also play a role in strengthening the therapeutic alliance between a patient and therapist during psychotherapy since the patient is more interested and open to social interaction. Moreover, it could be that by attenuating the perception of potentially or seemingly negative emotions that may be conveyed through the therapist’s facial expressions during psychotherapy, MDMA may promote a more positive and possibly safer space for the patient to address challenging emotions, such as the ones that may arise when talking about past traumatic events. Even if therapists do not actually express these negative facial expressions, patients can often project feelings of anger, fear and sadness onto the therapists^48^ and with the influence of MDMA this could be lowered to enable a safer space. Therefore, future studies should examine the specific interaction between therapist and patient in an MDMA assisted psychotherapy session to examine these hypotheses.

### Limitations

Firstly, the analysis of implicit emotional empathy included only three available studies, while other domains included 4 to 9 available studies. Secondly, this meta-analysis focused on studies in healthy volunteers, as there are no current studies investigating the effects of MDMA on these social functions in psychiatric populations. Acquiring this knowledge would enhance our comprehension of how MDMA influences individuals with psychiatric conditions, enabling us to tailor therapy more effectively, provide recommendations on who would benefit from it, and identify those who may be at risk of negative outcomes in therapeutic contexts.

It is important to note that these results point to a potential risk in using MDMA. When under the influence of MDMA, individuals may find themselves exceptionally open and responsive to the emotions and needs of others, often to the point of feeling highly connected. While this heightened empathic state may be therapeutic in a controlled setting, it also renders individuals potentially vulnerable to exploitation or abuse. The profound emotional openness induced by MDMA may lead individuals to trust and connect with others more readily, sometimes without appropriate discernment. This could be further exacerbated by a diminished ability to recognize and respond to negative emotions or potentially harmful situations, as MDMA can temporarily impair one’s ability to accurately interpret and act upon cues that signal danger such as an angry face. This heightened susceptibility highlights the critical importance of conducting MDMA-assisted therapy within a controlled and secure environment, under the guidance of trained professionals, who can provide the necessary support and ensure the safety and well-being of individuals undergoing this form of treatment. Additionally, comprehensive education and preparatory sessions for individuals participating in MDMA-assisted therapy can help them understand and navigate the potential risks associated with heightened empathy.

Unfortunately, the available data on mistakes made in the Facial Emotion Recognition Task (FERT) is notably lacking, which represents a significant gap in our understanding. This missing information is crucial, as it could have provided valuable insights into whether the decrease in accuracy when identifying negative emotions under the influence of MDMA may be attributed to a misattribution of these emotions as positive. Such an analysis would have offered another layer of insight into the underlying mechanisms contributing to these observed decreases in accuracy, shedding light on the specific cognitive processes affected by MDMA.

Another key limitation involves potential challenges with maintaining effective blinding. MDMA produces distinctive physiological and psychological effects including elevated heart rate, pupil dilation, and marked changes in mood and sociability that are often recognizable to both participants and experimenters^43^. Even under double-blind conditions, these overt cues can lead to partial unblinding and expectancy effects, potentially influencing both participants’performance on social-cognitive tasks and experimenters’subtle cues or interpretations. Several included studies attempted to mitigate this through active placebo controls (e.g., low-dose stimulants) or standardized instructions.

Additional limitation concerns heterogeneity in the measurement instruments and implementation of social-cognition tasks. While we restricted inclusion to studies using two well-validated paradigms—the Multifaceted Empathy Test (MET) and the Facial Emotion Recognition Task (FERT)—small procedural differences remained. These included variations in image sets, stimulus intensity gradients, rating scales, and whether arousal and concern ratings were analyzed separately or combined. Such differences can introduce subtle variability in task sensitivity and outcome comparability, which may contribute to between-study variance that is not fully captured by conventional heterogeneity statistics.

Finally, the current meta-analysis focuses exclusively on the *acute* effects of single-session MDMA administration. Although this design enhances experimental control and internal validity, it limits conclusions about the duration and functional significance of these effects. Longitudinal or follow-up data examining whether changes in empathy or emotion recognition persist after drug clearance remain sparse. Distinguishing between transient pharmacological modulation and longer-lasting neuropsychological change is critical, particularly for understanding MDMA’s potential role as an adjunct in psychotherapy. Future studies should therefore include repeated assessments to determine whether acute effects on social cognition translate into enduring therapeutic or behavioral outcomes.

### Study strengths and future directions

This meta-analysis is composed solely of randomized controlled trials that provide evidence for the effects of MDMA on certain social cognition functions. Statistical analyses revealed no publication bias and almost no heterogeneity in the studies included here, which has important implications for the interpretation and reliability of the findings. The observed lack of heterogeneity suggests that the studies included in this meta-analysis are consistent with each other and yield similar results or effect sizes. This was likely achieved here by focusing on single tasks for each construct of social cognition. These findings further suggest that any variability observed across the studies is likely due to random sampling error rather than differences in study characteristics or underlying population effects. The absence of heterogeneity strengthens the overall validity and generalizability of the meta-analytic results, suggesting that the findings are robust and that the effect being examined is relatively stable across the included studies.

We also did not rely on survey data in this meta-analysis and focused exclusively on behavioral data collected through the use of comparable computerized tasks (MET or FERT). There are several advantages to this approach. First, behavioral tasks provide objective and standardized measurements of participants’performance. Unlike self-report surveys, which rely on participants’subjective perceptions and interpretations, computerized tasks directly assess participants’behavior and responses. This objectivity reduces potential biases associated with self-reporting, leading to more accurate and reliable data. Second, computerized tasks lead to reduced demand characteristics as participants may consciously or unconsciously alter their responses in surveys based on social desirability or perceived expectations. In behavioral tasks, participants are typically less aware of the specific hypotheses being tested, which reduces demand characteristics, and increases the likelihood of natural and unbiased responses. This enables researchers to capture more objective effects of MDMA in a standardized controlled environment. This is particularly valuable for studying sensitive topics such as early or recent trauma or exploring unconscious processes that occur with psychoactive substances. Lastly, computerized tasks enable replicability and standardization as they can be easily replicated and standardized across different research settings and populations. Ultimately, all of these factors contribute to enhancing the generalizability of our findings.

The studies examined in this meta-analysis focused exclusively on a young participant cohort (all age ranges were between 18 and 38), underscoring the need for future research to expand its scope and investigate the effects of MDMA on a wider range of age groups. While our findings provide valuable insights into the impact of MDMA on empathy and emotion recognition in young individuals, extending these investigations to encompass children, adolescents, and middle-aged to elderly adults is essential. Such comprehensive studies would contribute to a more comprehensive understanding of the potential age-related nuances in the effects of MDMA on these crucial socioemotional domains. Moreover, while the current studies analyzed in this meta analysis did not reveal any significant sex differences in the effects of MDMA on empathy and emotion recognition, a closer examination of each sex individually was hindered by the limited sample sizes of each sex in each study (women: 9–16, men: 8–40). In order to comprehensively investigate the potential impact of MDMA on empathy and emotion recognition according to sex, it is imperative that future research endeavors prioritize larger and more balanced sample sizes for both genders.

Understanding and alleviating social cognition difficulties, particularly empathy and emotion recognition, holds significant importance in the context of psychiatric disorders. Empathy deficits are commonly observed in various psychiatric conditions such as depression^49^, addiction^50^, borderline personality disorder^51^, bipolar disorder^52^ and psychopathy^53^. These disorders are broadly characterized by impaired interpersonal functioning and difficulties in relating to others. By investigating the effects of interventions like MDMA on empathy and facial expression recognition, we can gain valuable insights into potential therapeutic avenues for addressing these deficits. Enhancing social cognition abilities has the potential to improve interpersonal relationships, communication skills, and overall quality of life for individuals affected by these disorders. Moreover, it can contribute to the development of targeted interventions that focus on restoring and promoting empathic abilities in clinical populations. Therefore, acquiring a deeper understanding of the mechanisms underlying the enhancement of empathy is important for advancing the field of mental health and developing novel interventions for psychiatric disorders.

## Conclusion

In conclusion, this meta-analysis provides valuable insights into the effects of MDMA on social cognition. The results suggest that MDMA administration, compared to placebo, enhances emotional empathy while concurrently diminishing the recognition of negative facial expressions like sadness, fear, and anger. The analysis did not find significant effects of MDMA on cognitive empathy or the recognition of happy facial expressions. These findings suggest that MDMA may have complex and multifaceted effects on social cognition and underscore the need for further research to clarify the underlying psychological and neural mechanisms. Understanding the specific effects of MDMA on different aspects of social cognition is crucial for optimizing its application as a therapeutic adjunct and ensuring the safety and efficacy of its use in clinical settings. Future studies should delve into these nuanced effects to inform research on MDMA-assisted psychotherapy and the development of novel treatments for disorders associated with deficits in social functioning.

## Data Availability

The datasets used and/or analyzed during the current study are available from the corresponding author on reasonable request.
